# Mad River Valley Dental Society

**Published:** 1874-08

**Authors:** A. J. Grosvenor

**Affiliations:** Sec.


### MAD RIVER VALLEY DENTAL SOCIETY.

The Mad River Valley Dental Society met in annual session at the Phillips House, Dayton, O., on Tuesday, April 7, 1874, at 11 o'clock a. m.

President, Dr. Keeley, in the chair.

Meeting opened with prayer by Dr. Geo. Watt. Minutes of last meeting read and approved. The morning session was occupied in organizing and getting into working order.

Dr. Taft reported the name of Dr. J. C. Oldham for membership, and he was duly elected. Adjourned until 2 p. m.

President Keeley called the Society to order at 2 p. m. Reading of essays and discussion of topics were taken up. Dr. Tizzard read an essay on "Abnormal Mucus Membrane." The first subject and essay having the same general bearing, on motion, they were taken up and discussed as one.

Dr. Taft exhibited a number of new appliances which were examined with great interest. Adjourned to 7:30 p. m.

At 7:30 discussions resumed and continued until 10 p. m.

All present seemed deeply interested in the discussions and proceedings, and felt amply paid for coming. The attendance of resident dentists was larger than ever before. The President, Dr. Keeley, appointed as Executive Committee, Drs. C. Bradley, Dayton; A. Berry, Cincinnati; and A. J. Grosvenor, Troy. Essayists, Drs. R. H. Boal, A. A. Blount, J. C. Oldham and H. A. Smith. Delegates to the American Dental

Society, at Detroit, Aug. 4th, 1874, Drs. C. Bradley, George Watt, S. B. Tizzard, A. J. Grosvenor, E. F. Sample and J. C. Oldham.

The Secretary was instructed to issue certificates to each delegate.

On motion, all money in the treasury on August 1st, 1874, was ordered to be paid to the committee appointed for that purpose by the American Dental Society, as a slight testimonial to Dr. Barnum, the inventor of Rubber-Dam, for his gift to the profession.

The election of officers for the ensuing year resulted as follows: President, Dr. S. B. Tizzard; Vice-President, Dr. E. F. Sample; Secretary and Treasurer, Dr. A. J. Grosvenor.

On motion, adjourned to meet in Dayton, on Thursday, October 6th, 1874, in semi-annual session.

A. J. Grosvenor, Sec.

